# Protocol for detecting genomic insulators in *Drosophila* using insulator-seq, a massively parallel reporter assay

**DOI:** 10.1016/j.xpro.2024.103391

**Published:** 2024-10-24

**Authors:** Anastasiia Tonelli, Pascal Cousin, Maria Cristina Gambetta

**Affiliations:** 1 Center for Integrative Genomics, University of Lausanne, 1015 Lausanne, Switzerland

**Keywords:** Sequencing, RNA-seq, Model Organisms, Gene Expression

## Abstract

Genomic insulators are DNA elements that prevent transcriptional activation of a promoter by an enhancer when interposed. We present a protocol for insulator-seq that enables high-throughput screening of genomic insulators using a plasmid-based massively parallel reporter assay in *Drosophila* cultured cells. We describe steps for insulator reporter plasmid library generation, transient transfection into cultured cells, and sequencing library preparation and provide a pipeline for data analysis.

For complete details on the use and execution of this protocol, please refer to Tonelli et al.^1^

## Highlights

• Instructions for high-throughput screening of genomic insulators in *Drosophila* cells
• Steps for high-throughput barcoded insulator reporter plasmid library generation
• Guidance on DNA- and RNA-seq library preparation and quality control

## Abstract (editor highlights)

Publisher’s note: Undertaking any experimental protocol requires adherence to local institutional guidelines for laboratory safety and ethics.

## Graphical abstract

**Figure:**
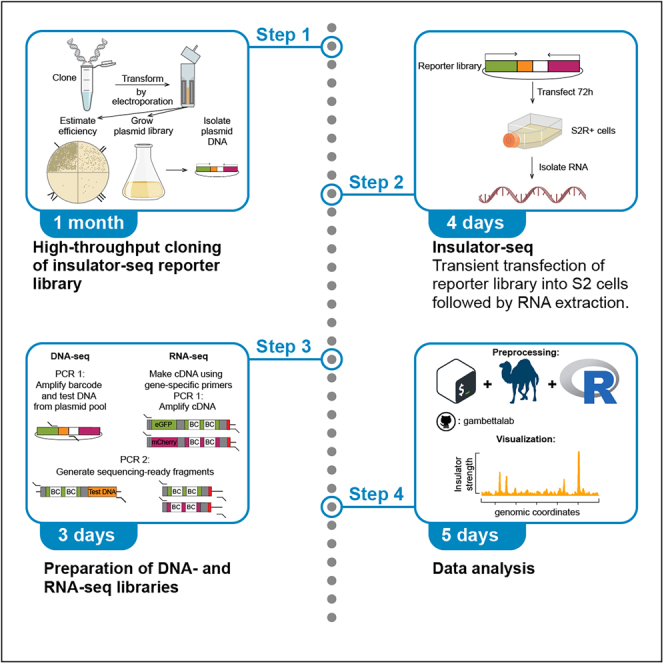

## Before you begin

### Barcode oligo pool design

**Timing: 1–2 days**

Efficient barcoding of the insulator reporter library is crucial for the success of insulator-seq. When designing the barcode library, our requirements were the following: (1) The complexity of the barcode library must greatly exceed the complexity of cloned test fragments. (2) Barcodes must not have long stretches of nucleotides or inverted repeats. (3) Barcodes must remain distinguishable in the case of 1 nucleotide mismatch. (4) Barcodes must not contain recognition sites of restriction enzymes used to generate the reporter library. To create the barcode library, we used the Bioconductor package DNABarcodes.^2^ Requirements (2) and (3) are met with the standard parameters of the create.dnabarcodes function of the package. Since the number of possible barcodes is directly proportional to the sequence length, we used 12 bp barcodes, which gives approximately 90000 usable barcodes.

Our cloning strategy allowed us to combine random pairs of these barcodes (see “high-throughput cloning of insulator-seq library” section for details), increasing the maximum theoretically possible diversity to more than 5 billion barcodes, which guaranteed diversity of the acceptor library and met requirement (1). To meet requirement (4), we used bash commands to remove undesired sequences. Step-by step instructions and code used to generate the reporter barcode oligo pool are shown below.

1. Set working directory, download and load the required packages.

#Set working directory

setwd("")

#Download required packages

if (!require("BiocManager", quietly = TRUE))

 install.packages("BiocManager")

BiocManager::install("DNABarcodes")

#Load the required packages

library(DNABarcodes)

library(tidyverse)

2. Generate the barcodes of desired length.

# Generate the barcode of desired length

my_barcodes <- data.frame(create.dnabarcodes(12))

***Note:*** We were unable to generate barcodes longer than 10 bp on a regular computer (1.4 GHz Quad-Core Intel Core i5, 16 GB RAM) and therefore generated the 12 bp barcode list on a cluster.

3. Reverse complement the barcodes.

# Rename the first column

colnames(my_barcodes) <- c("seq")

# Convert factor to character

my_barcodes$seq <- as.character(my_real_barcodes$seq)

#Make a function for reversing a string

strReverse <- function(x)

 sapply(lapply(strsplit(x, NULL), rev), paste, collapse="")

# Reverse complement the barcodes

my_barcodes$seq_rv <-strReverse(chartr('ATGC', 'TACG', my_barcodes$seq))

**Figure 1:**
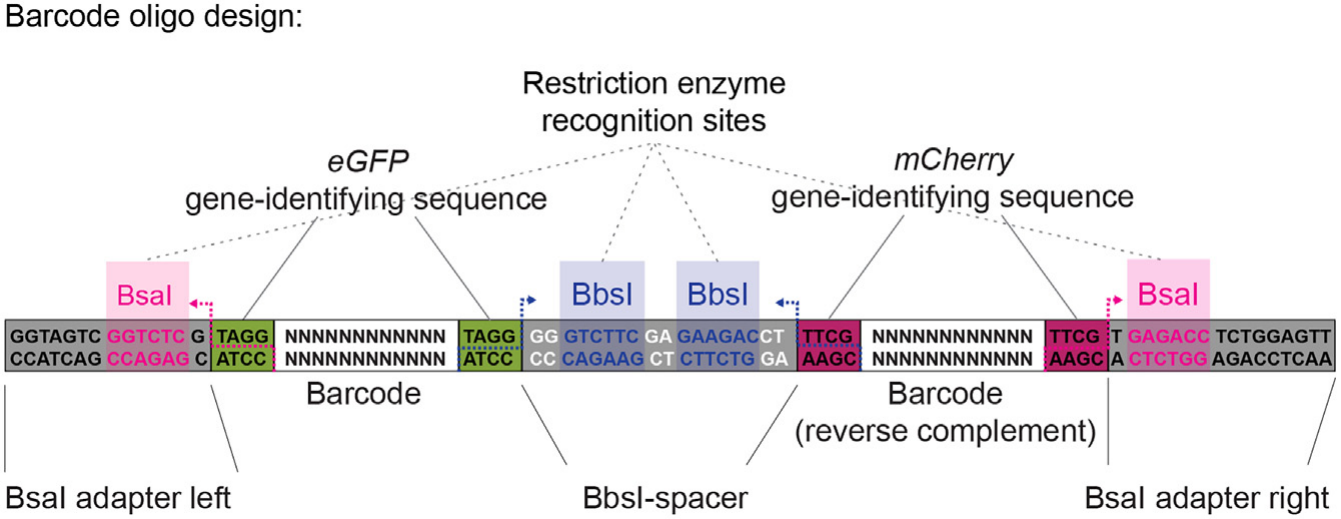
Barcode oligo design The parts of the insulator-seq barcode oligos are shown. The color code used for gene-identifying sequences was kept in all figures.

4. Create gene-identifying sequences (Figure 1), BbsI spacer between the barcodes, and left and right BsaI adapter sequences as part of the same data frame, then combine them into one string in the correct order.

# Add gene tag sequences

my_barcodes$GFP <- "TAGG"

my_barcodes$mCherry <- "TTCG"

#Add spacer BbsI-containing spacer between the barcodes

my_barcodes$BbsI_spacer <-"GGGTCTTCGAGAAGACCT"

# Add left and right BsaI adapters

my_barcodes$BsaI_left <- "GGTAGTCGGTCTCG"

my_barcodes$BsaI_right <- "TGAGACCTCTGGAGTT"

# Assemble barcode into one string

my_barcodes$final <- as.character(do.call(paste0, my_barcodes[c("BsaI_left", "GFP", "seq", "GFP","BbsI_spacer","mCherry", "seq_rv", "mCherry", "BsaI_right")]))

5. Remove barcodes that contain restriction enzyme recognition sites used in insulator-seq acceptor library cloning.

#Remove restriction enzyme sites.Remember to remove sites on both strands

restriction_enzyme_sites <- c('GGTCTC.∗GGTCTC', 'GAGACC.∗GAGACC', 'GTCTTC.∗GTCTTC', 'GAAGAC.∗GAAGAC', "GGATCC", "AATAAA", "TTTATT")

my_barcodes_final <- my_barcodes[grep(paste(restriction_enzyme_sites, collapse = "|"), my_barcodes$final, invert = TRUE),]

6. Save the final table with the barcode oligo list.

### Synthetic insulator oligo pool design

**Timing: 1–2 days**

This step is optional and only required for targeted screening of a select list of designer test fragments. Below we provide a step-by-step pipeline that is intended to serve as a guide for designing a synthetic oligo pool that could be cloned into the insulator-seq acceptor library.

7. Select fragments of interest.

***Note:*** For the synthetic insulator screen in our study, we focused on sites bound by CTCF, a conserved insulator-binding protein associated with enhancer-blocking insulators and topological domain boundaries in flies and mammals.^3^ We selected CTCF motifs (JASPAR motif MA0531.1) found under CTCF ChIP-seq peaks identified in our previous study,^4^ and the test fragments were centered on the motif.

8. Choose the size of the fragment.

***Note:*** The size of test fragments is limited by the cost of oligo pool synthesis. In our study, we chose the test fragments to be 190 bp long.

***Optional:*** Design mutagenized versions of the wildtype fragments of interest. An example of how to design motif point mutations is available on GitHub.^5^

***Optional:*** Add a barcode identifier to the test sequence.

***Note:*** In our study this was necessary to distinguish between mutagenized fragments that differed from other sequences by single base pair substitutions. We placed a 6 bp barcode at the 5′ end of each test fragment. For instructions on how to generate a barcode, see code block above.

9. Add adapter sequences at the 5′ and 3′ ends for oligo pool amplification by PCR.

***Note:*** In our study, we appended the same sequence (TGTAAAACGACGGCCAG) in 5′ and 3′ of the inserts to allow bidirectional cloning.

10. Each final fragment in the oligo pool library had the following structure: [TGTAAAACGACGGCCAG]-[6 bp barcode]-[190 bp test fragment]-[CTGGCCGTCGTTTTACA].
11. Design primers to PCR-amplify the oligo pool library and add Gibson homology arms.

**Table 1:** PCR Primers and DNA oligos used for insulator-seq

Name	Sequence (5′>3′)	Purpose
Tn5ME rv	[Phos]-CTGTCTCTTATACACATCT	Tn5 tagmentation
Tn5ME-GibsonL15	GCAATAAACAAGTTGAGATGTGTATAAGAGACAG	Tn5 tagmentation
Tn5ME-GibsonR15	GTTTATCATCATGGGAGATGTGTATAAGAGACAG	Tn5 tagmentation
Tn5ME-GibsonL PCR	AAGCTGCAATAAACAAGTTGAGATGTG	Tn5 tagmentation
Tn5ME-GibsonR PCR	TACATTGTTTATCATCATGGGAGATGTG	Tn5 tagmentation
BC fw	GGTAGTCGGTCTCGTAGG	barcode library amplification
BC rv	AACTCCAGAGGTCTCACG	barcode library amplification
Gibson Homology M13 fw	AAGCTGCAATAAACAAGTTGTGTAAAACGACGGCCAG	synthetic insulator amplification
Gibson Homology M13 rv	TACATTGTTTATCATCATGGGTGTAAAACGACGGCCAG	synthetic insulator amplification
DNA PCR 1 fw	TCGTCGGCAGCGTCAGATGTGTATAAGAGACAGGA AATTTGTGATGCTATTTAGG	DNA-seq library preparation
DNA PCR 1 rv	GTCTCGTGGGCTCGGAGATGTGTATAAGAGACAG CATACATTGTTTATCATCATGGG	DNA-seq library preparation
RNA PCR 1 fw	AAGCCACCATGGAAAAGGCCA	RNA-seq library preparation
UMI RT primer	TCGTCGGCAGCGTCAGATGTGTATAAGAGAC AG[8N UMI identifier]GCAGCTTATAAT GGTTACAAATAAAGC	UMI-RNA-seq library preparation
DNA/RNA PCR 2 fw	AATGATACGGCGACCACCGAGATCTAC AC[6N Illumina barcode] TCGTCGGC AGCGTCAGATG	DNA/RNA-seq library preparation
DNA/RNA PCR 2 rv	CAAGCAGAAGACGGCATACGA GAT[6N Illumina barcode] GTCTC GTGGGCTCGGAGATG	DNA/RNA-seq library preparation

***Note:*** In our study, we used M13 fw and M13 rv (see Table 1 for sequences) as 5′ and 3′ Gibson homology sequences, respectively.

## Key resources table

**Table:** 

REAGENT or RESOURCE	SOURCE	IDENTIFIER
**Bacterial and virus strains**		

DH5a	NEB	C2987H
pirHC	Geneva Biotech	N/A

**Chemicals, peptides, and recombinant proteins**		

Effectene transfection reagent	QIAGEN	301427
TRIzol LS reagent	Ambion	10296028
BsaI-HF-v2	NEB	R3733L
BbsI	Thermo Fisher Scientific	ER1012
BamHI-HF	NEB	R3136S
T4 ligase	Thermo Fisher Scientific	EL0016
SuperScript IV	Thermo Fisher Scientific	18090050
TURBO DNase	Thermo Fisher Scientific	AM2238
Tn5	Hennig et al.^6^	N/A

**Critical commercial assays**		

PureYield plasmid midiprep system	Promega	A2495
MinElute PCR purification kit	QIAGEN	28004
NucleoSpin gel and PCR clean-up	Macherey-Nagel	740609.50
QIAGEN plasmid midi kit	QIAGEN	12124
KAPA HiFi PCR kit	Roche	7958838001
KAPA HiFi HotStart ReadyMix	Roche	07958935001
Qubit dsDNA HS assay kit	Thermo Fisher Scientific	Q32854
Qubit RNA HS assay kit	Thermo Fisher Scientific	Q32852

**Deposited data**		

Insulator-seq data	Tonelli et al.^1^	GEO: GSE253140

**Experimental models: Cell lines**		

*Drosophila* S2R+ cells	Drosophila Genomics Resource Center	DGRC Cat# 150, RRID:CVCL_Z831

**Oligonucleotides**		

The list of oligos is provided in Table 1	N/A	N/A

**Recombinant DNA**		

D. melanogaster genomic DNA covering BX-C	BACPAC Resources Center	CH321-80H22; CH321-60D22; CH321-68P11; CH321-22B19.
insulator_seq_barcode_vector	Addgene	221458
insulator_seq_UENU_donor	Addgene	221459
insulator_seq_reporter_vector	Addgene	221460

**Software and algorithms**		

Custom code	Tonelli et al.^1^^,^^5^	https://github.com/gambettalab/Insulator-seq-2024
DNA Barcodes v.1.34.0	Buschmann and Bystrykh^2^	https://doi.org/10.18129/B9.bioc.DNABarcodes
Bowtie2 v.2.4.5	Langmead and Salzberg^7^	https://bowtie-bio.sourceforge.net/bowtie2/index.shtml
ggplot2 v.3.3.6	Wickham^8^	https://ggplot2.tidyverse.org/
Bedtools v.2.31.1	Quinlan et al.^9^	https://bedtools.readthedocs.io
Gviz v.1.48.0	Hahne and Ivanek^10^	https://doi.org/10.18129/B9.bioc.Gviz

**Other**		

Thermocycler	N/A	N/A
1.5 mL DNA LoBind tube	Eppendorf	022431021
RNAClean XP	Beckman-Coulter	A63987
SPRIselect	Beckman-Coulter	B23318
DynaMag-2 magnetic stand	Invitrogen	12321D
Fluorometer	DeNovix	QFX
Bioanalyzer	Agilent	2100
Gene Pulser Xcell total system	Bio-Rad	1652660
Gene Pulser cuvette	Bio-Rad	1652086

## Materials and equipment

**Table:** Agarose desalting filters (modified from Atrazhev and Elliott^11^)

Reagent	Final concentration	Add for 50 mL
Glucose	100 mM	1.9 g
Agarose	2% (w/v)	2 g
ddH_2_O	–	Up to 50 mL

Mix the powders in water and heat until dissolved. Distribute 2 × 600 μL into 1.5 mL tubes using a cut 1000 μL tip. The tube should not be filled completely. Add 200 μL pipette tips to the tubes, setting them straight. The end of the tip should not be touching the bottom of the tube. Wait until agarose hardens. Remove the pipette tips, close the tubes. Store at 4°C for up to 2 weeks.

**Table:** SOC recovery medium

Reagent	Stock concentration	Final concentration	Add to 800 mL
BactoTryptone	–	0.02% (w/v)	20 g
Yeast extract	–	0.005% (w/v)	5 g
NaCl	–	8.5 mM	0.5 g
KCl	1 M	2.5 mM	2 mL
ddH_2_O	–	–	Up to 970 mL
Glucose	20% (w/v)	0.4%	–
MgCl_2_	1 M	1 mM	–

Dissolve tryptone, yest extract and NaCl in 800 mL of ddH_2_O and add 2.5 mL KCl. Adjust pH to 7.0 and fill to a final volume of 970 mL. Aliquot in 10 × 97 mL aliquots. Sterilize by autoclaving. Immediately before use, add 1 mL of 1M MgCl_2_ and 2 mL of 20% glucose.

### Electrocompetent cells and transformation by electroporation

We prepared electrocompetent pirHC cells as described in the published step-by-step protocol.^12^ Competent cells were split into 350 μL aliquots.

Cells were electroporated using the exponential decay protocol on a Gene Pulser Xcell Total System, using 2 mm cuvettes with the following settings.^13^

**Table:** 

Voltage	2400 V
Capacitance	25 μF
Resistance	750 Ω
Cuvette	2 mm

## Step-by-step method details

**Figure 2:**
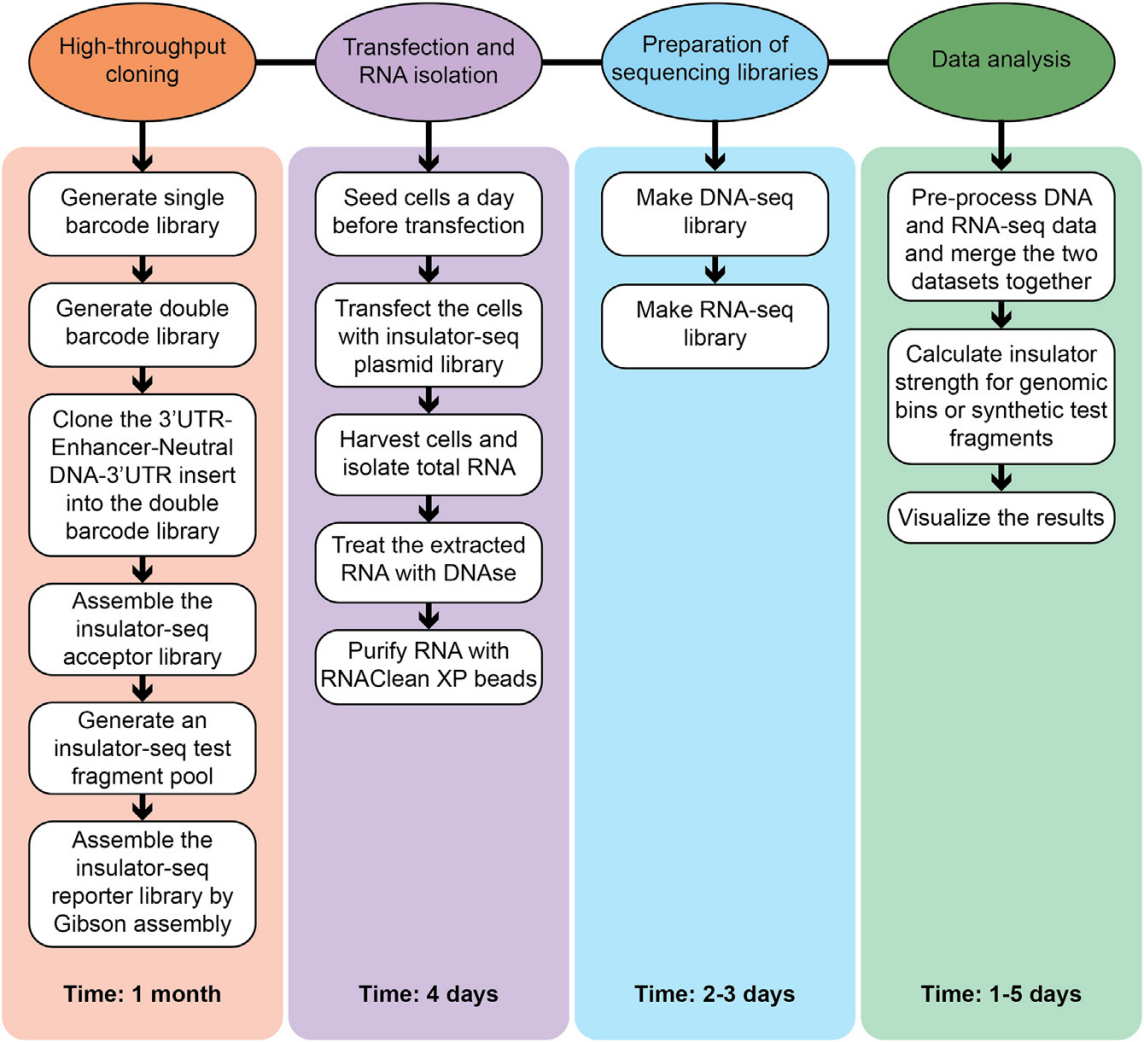
Overview of the major protocol steps The flowchart shows the main steps and the substeps of the insulator-seq protocol.

An overview of all steps of the insulator-seq protocol is provided in Figure 2.

### High-throughput cloning of insulator-seq library

**Timing: 1 month**

**Figure 3:**
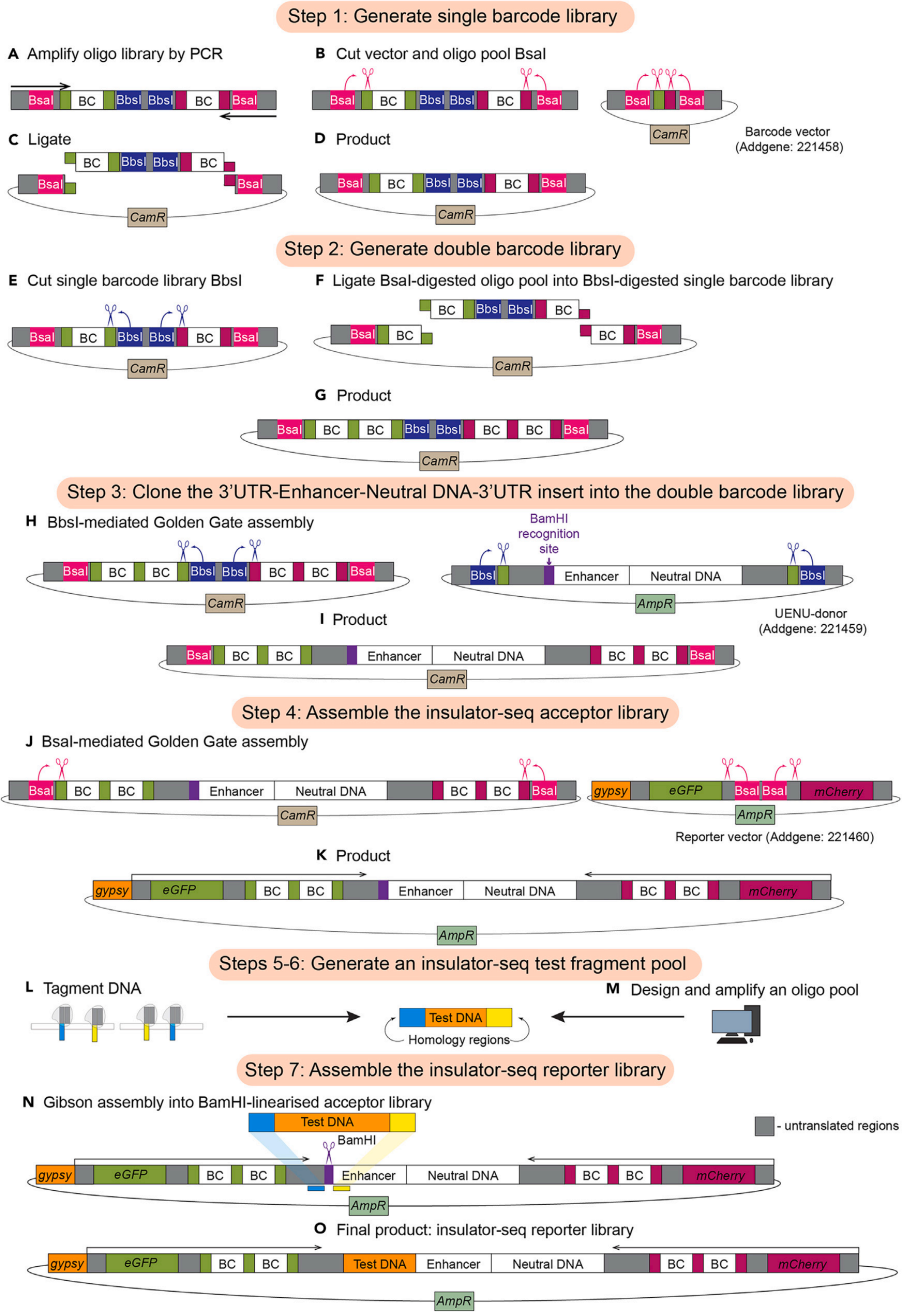
Insulator-seq reporter library cloning (A) Barcode oligo pool is amplified by PCR (step 1a). (B) Amplified barcode oligo pool and vector (insulator-seq barcode vector; Addgene 221458) are digested with BsaI (step 1b). (C) BsaI-digested barcode oligo pool and vector are ligated, preserving BsaI-recognition site (step 1c). (D) Result of cloning step 1: single barcode plasmid (step 1d). (E) Single barcode plasmid pool is digested with BsaI (step 2a). (F) BsaI-digested barcode oligo pool and BbsI-digested single barcode plasmid pool are ligated. (G) Result of cloning step 2: Double barcode plasmid pool. (H) 3′UTR-Enhancer-Neutral DNA-3′UTR sequence (Addgene 221459) is inserted between the double barcodes by BbsI-mediated Golden Gate assembly (step 3a). (I) Result of cloning step 3: double barcodes separated by the enhancer, neutral DNA, parts of 3′UTR sequences. (J) Double barcode and enhancer insert pool is inserted between the reporter genes (Addgene 221460) by BsaI-mediated Golden Gate assembly (step 4a). (K) Result of cloning step 3: insulator-seq acceptor library. (L) Insulator-seq insert is generated by tagmenting DNA of the locus of interest (step 5). (M) Insulator-seq insert is generated by PCR-amplifying designer oligo pool library (step 6). (N) Insulator-seq insert is assembled into BamHI-linearized insulator-seq acceptor library by Gibson assembly (step 7). (O) Product of cloning step 7 – insulator-seq reporter library.

This step encompasses the entire insulator-seq plasmid library generation process (Figure 3). Common procedures to all cloning steps are described in detail in step 1, and we advise to follow the same procedure for desalting, transformation and estimating cloning efficiency for the subsequent cloning steps.

***Note:*** Before proceeding with the large-scale cloning reactions described in this section, we recommend first cloning the spike-in controls, at small-scale. Spike-in controls are reporters with insulators of known strengths. We included 4 control insulators in our experiments: neutral DNA lacking insulator activity, the well-characterized and potent *gypsy* insulator, and two insulators of intermediate strengths (CTCF-bound fragments “B” and “N” described in our previous study^4^). The spike-in controls provide a preliminary validation to ensure that the reporter assay is working as expected but they are not used in the downstream analysis. Unique barcode sequences that are not part of the synthesized barcode oligo pool (described in the “Before you begin” section) are used for generating spike-in controls.

1. Generate single barcode library.
  a. Amplify the oligo pool library (Figure 3A).
    i. Set up a PCR reaction on ice:

**Table:** 

Reagent	Amount
DNA template	500 ng
KAPA HiFi HotStart ReadyMix	250 μL
Primer 1 (BC fw)	1 mM
Primer 2 (BC rv)	1 mM
ddH_2_O	to 500 μL
    ii. Mix by pipetting up and down, centrifuge briefly.
    iii. Split the master mix into 20 tubes.
    iv. Start the PCR program on a thermocycler with a lid heated to 110°C, and transfer tubes from ice into the thermocyler when the block reaches 98°C:

**Table:** 

Steps	Temperature	Time	Cycles
Initial Denaturation	98°C	45 s	1
Denaturation	98°C	15 s	8–10 cycles
Annealing	64°C	30 s	
Extension	72°C	15 s	
Final extension	72°C	1 min	1
Denature	95°C	15 s	1
Cool down	−0.5°C	15 s	140 cycles
Hold	10°C	Forever

      ***Note:*** We recommend optimizing the number of cycles (8–10 cycles), amount of template and volume of master mix, such that the lowest number of cycles is used to generate a sufficient amount of product while maintaining a 2–5-fold amplification relative to the input.
      ***Note:*** Emulsion PCR may lead to better results, as discussed in the “limitations” section.
  b. Purify the PCR product with a MinElute PCR Purification Kit (QIAGEN) or equivalent.
    i. Pool the PCR reactions into one tube.
    ii. Add 5 volumes of PB buffer without the pH indicator to 1 volume of PCR reactions.
    iii. Add 10 μL of 3 M sodium acetate, pH 5.0.
    iv. Vortex to mix.
    v. Apply 750 μL sample to the MinElute column. Centrifuge 12000 g for 1 min, discard supernatant and repeat until all the sample has gone through the column.
    vi. To wash the column, add 750 μL Buffer PE, centrifuge 12000 g for 1 min and discard supernatant. Wash the column 3 times.
    vii. To remove residual ethanol, centrifuge the column 12000 g for 5 min.
    viii. Place the column over a clean 1.5 mL DNA LoBind tube and let ethanol evaporate by incubating an open column 10 min at 20°C–25°C.
    ix. To elute DNA, add 15 μL of elution buffer (10 mM Tris-HCl pH 8.5) to the column. Let the column stand for 1 min, then centrifuge 12000 g for 1 min. Repeat the elution twice, so that the total volume of elution buffer added to the column is 30 μL.
    x. Measure the concentration of eluted DNA by using Qubit dsDNA HS assay kit (Thermo Fisher Scientific) or equivalent. Expect 1 μg total DNA yield from 500 μL PCR reaction.
      **Pause point:** Purified PCR product can be stored at −20°C.
  c. Inoculate insulator-seq barcode vector-carrying bacteria (Addgene 221458) into 50 mL of LB with selective antibiotic (34 μg/mL chloramphenicol) and incubate at 37°C 16–18 h with vigorous shaking in a bacterial incubator.
  d. Isolate vector plasmid DNA from the *E. coli* cultures using PureYield Plasmid Midiprep System (Promega) or equivalent.
  e. Digest vector and insert (PCR product from step b) with BsaI and purify the digestion product (Figure 3B).
    i. Set up vector digestion:

**Table:** 

Reagent	Amount
Vector (pIDC screen) plasmid DNA	4 μg
10× rCutSmart Buffer	20 μL
BsaI-HF-v2	4 μL
ddH_2_O	To 200 μL
    ii. Incubate 3h at 37°C in a thermocycler with a heated lid.
    iii. Set up insert digestion:

**Table:** 

Reagent	Amount
Barcode oligo pool PCR product	1 μg
10× rCutSmart Buffer	10 μL
BsaI-HF-v2	1 μL
ddH_2_O	To 100 μL
    iv. Incubate 1 h at 37°C in a thermocycler with a heated lid.
      **CRITICAL:** Do not over-digest vector and insert. Longer incubation times lead to decrease in cloning efficiency.
    v. Purify vector and insert digestion products using NucleoSpin Gel and PCR Cleanup kit (Macherey-Nagel) or equivalent. Column purification is better suited for this step than gel extraction.
    vi. Measure DNA concentration of digested DNA. Expect to yield 60%–80% of input DNA.
      **Pause point:** Purified digestion products can be stored at −20°C.
  f. Ligate vector and insert (Figures 3C and 3D).
    i. Set up ligation reaction.

**Table:** 

Component	Final amount
Vector – 1838 bp	600 ng
Insert – 88 bp	30 ng (equimolar to vector)
T4 ligase buffer	6 μL
T4 ligase	2 μL
ddH_2_O	To 60 μL

      ***Note:*** For high throughput cloning reactions, we recommend not ligating more than 1 μg of total DNA per reaction. Unless otherwise indicated, we ligated equimolar amount of vector and insert.
    ii. Include a negative control reaction without the insert.
    iii. Incubate 1 h at 20°C in a thermocycler.
  g. Desalt the ligation reaction.
    i. Prepare the agarose filters as described in the materials and equipment section and shown in Figure 4.

**Figure 4:**
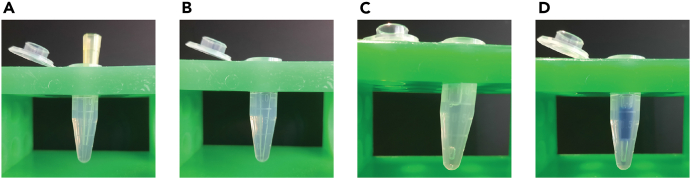
Agarose filter preparation and use (A) Agarose filter with a pipette tip inside after agarose hardened. (B) Empty well created by removing the pipette tip after agarose hardened. (C) Sample loaded onto the agarose filter. Note the air pocket under the sample. (D) Same as C, but the sample was colored to help visualize the correct position of the sample in the filter. We do not recommend staining the sample for the experiment.
    ii. Hold the open tube with the agarose filter at 45° angle.
    iii. Apply 60 μL of cloning reaction to the center of the agarose well. Close the tube carefully.
      **CRITICAL:** Avoid that the sample falls to the bottom of the agarose filter tube.
    iv. Incubate on ice for 30–45 min.
      ***Optional:*** Transfer the desalted reaction to a clean tube and keep on ice until transformation.
      ***Note:*** Desalting on an agarose filter will decrease the sample volume. When loading 60 μL of cloning reaction, we usually recover 40–50 μL.
  h. Transform the desalted cloning reaction into electrocompetent *E. coli.*
    i. Remove an aliquot of frozen electrocompetent cells from the freezer and thaw on ice for 10–15 min.
    ii. Place electroporation cuvettes on ice to precool.
    iii. Aseptically transfer 25 mL of SOC medium (see materials and equipment) into a 250 mL Erlenmeyer flask.
    iv. Place a 1 mL sterilized glass Pasteur pipette with a bulb fitted into the flask with SOC medium.
    v. Turn on the electroporator (Gene Pulser Xcell Total System, Bio-Rad) and enter the settings indicated in the materials and equipment section.
      **CRITICAL:** Steps ii-v above should be completed while the cells are thawing. Use the thawed cells as soon as possible, and do not keep them on ice for longer than 30 min.
    vi. Add 50 μL of desalted cloning reaction to 350 μL of thawed electrocompetent cells.
    vii. With a micropipette set to 350 μL, carefully mix by pipetting up and down 3–5 times. Avoid air bubbles.
    viii. Remove the cap from the electroporation cuvette and carefully pipette the cells and DNA mix onto the side of the cuvette, holding it at an angle. Do not completely fill the cuvette and discard the excess mix.
    ix. Place the cuvette on ice and put on the cap. Avoid splashes and bubbles on the sides of the cuvette.
    x. Quickly but thoroughly dry the cuvette with tissue paper before placing it into the pod. Press the “pulse” button immediately after locking the lid.
      Troubleshooting 1**:** An arc appears during electroporation.
    xi. In case of successful electroporation, expect the time constant to be between 4-10 ms.
    xii. Immediately after successful electroporation is confirmed, aspirate 1 mL of SOC medium with the Pasteur pipette, remove cuvette from the pod, and add the medium to the cuvette. Immediately aspirate the cells and slowly transfer them to the flask. Aspirate SOC medium from the part of the flask the cells haven’t reached and repeat a total of 4 times maximum. This helps quantitatively recover the cells from the cuvette.
    xiii. Gently shake the flask to distribute the cells.
    xiv. Let the cells recover by incubating the preculture at 37°C for 30 min with gentle shaking (160 rpm) in a bacterial incubator.
  i. Plate serial dilutions of recovered cells to estimate transformation efficiency.
    i. Remove 50 μL aliquot from the preculture.
    ii. Make 3 10-fold serial dilutions using SOC medium without selective antibiotic (1:10, 1:100, 1:1000).
    iii. Divide a petri dish with LB-agar and selective antibiotic into equal quarters.
    iv. Plate 5 μL of undiluted preculture and 5 μL of each serial dilution onto a quarter of the petri dish using a micropipette tip to distribute the liquid.
    v. Incubate 16–18 h at 37°C.
  j. Inoculate cultures of transformed library.
    i. Add 225 mL of LB with selective antibiotic into a 2 L Erlenmeyer flask.
    ii. Add 25 mL of preculture.
    iii. Incubate 12–16 h at 37°C with gentle shaking (160 rpm) in a bacterial incubator.
  k. Estimate the transformation efficiency.
    i. Count the number of colonies in the quadrants where it is possible.
    ii. Estimate transformation efficiency using the following calculations: 1 colony in undiluted quadrant = 5·10^3^ colonies in the preculture, 1 colony in the first dilution = 5·10^4^ in the preculture, 1 colony in the second dilution = 5·10^5^ in the preculture, 1 colony in the third dilution = 5·10^6^ in the preculture.
    iii. Estimate the background by comparing number of colonies in the negative control and ligation transformations.
      Troubleshooting 2**:** Low transformation efficiency or no colonies at all.
  l. Isolate plasmid DNA from 50 mL of culture using PureYield Plasmid Midiprep System (Promega) or equivalent.
    ***Note:*** The remaining 200 mL of bacterial can be aliquoted, centrifuged and stored as frozen bacterial pellets at −20°C or −80°C.
    **Pause point:** Plasmid DNA can be stored at −20°C.
2. Generate double barcode library (Figures 3E–3G).
  ***Note:*** This step greatly increases the diversity of barcodes (up to 10 billion possible combinations of ∼100′000 unique single barcodes) by creating random pairs of barcodes. In principle, this step can be repeated to generate triple barcodes and so on, but we have never obtained the number of colonies that would reach the theoretically possible number of barcode combinations. For generating spike-in controls, we purposefully skipped this step, and generated reporters that only have single barcodes.
  a. Digest single barcode plasmid library with BbsI.
    i. Set up digestion reaction:

**Table:** 

Reagent	Amount
Vector plasmid DNA	4 μg
10× Buffer G	20 μL
BbsI	4 μL
ddH_2_O	To 200 μL
    ii. Incubate 3h at 37°C in a thermocycler.
    iii. Purify the digestion product with a NucleoSpin Gel and PCR Cleanup kit (Macherey-Nagel) or equivalent. Gel extraction is better suited for this step than column purification.
    iv. Measure DNA concentration of digested DNA. Expect to yield 60%–80% of input DNA.
      **Pause point:** Purified digestion products can be stored at −20°C.
  b. Ligate BsaI-digested barcode PCR (from step 1a) into the BbsI-digested single barcode library (from step 2a).
    i. Set up ligation reaction:

**Table:** 

Component	Final amount
Vector – 1884 bp	600 ng
Insert – 88 bp	30 ng (equimolar to vector)
T4 ligase buffer	6 μL
T4 ligase	2 μL
ddH_2_O	To 60 μL
    ii. Incubate 1 h at 20°C in a thermocycler.
  c. Desalt the ligation reaction as described in step 1g.
  d. Transform the desalted reaction into electrocompetent *E. coli* as described in step 1h.
  e. Plate serial dilutions of recovered cells to estimate transformation efficiency as described in 1i.
  f. Inoculate cultures of transformed library as described in step 1j.
  g. Estimate transformation efficiency as described in 1k and double barcode diversity.
    ***Note:*** The diversity of double barcode combinations that can be obtained vastly exceeds the number of colonies obtained. Therefore, the number of colonies obtained during this step is a direct estimate of the diversity of the double barcode library. The number of colonies in the negative control is an estimate of how many single barcodes remain in the double barcode pool. If a large number of colonies is obtained in the negative control (>5% of the number of colonies obtained in the double barcode cloning reaction), we recommend optimizing the vector digestion step. Note that single barcodes will not be removed in subsequent cloning steps, unlike the no-insert vector that will not be transferred past this step.
  h. Isolate plasmid DNA from the 50 mL culture using a PureYield Plasmid Midiprep System (Promega) or equivalent.
    **Pause point:** Plasmid DNA can be stored at −20°C.
3. Clone the 3′UTR-Enhancer-Neutral DNA-3′UTR insert into the double barcode library.
  a. Inoculate insulator-seq UENU donor-carrying bacteria (Addgene 221459) into LB with selective antibiotic (100 μg/mL ampicillin) and incubate at 37°C 16–18 h with vigorous shaking in a bacterial incubator.
  b. Isolate plasmid DNA from the *E. coli* culture using PureYield Plasmid Midiprep System (Promega) or equivalent.
  c. Use the double barcode library obtained in the previous step as a vector. The 3′UTR-Enhancer-Neutral DNA-3′UTR sequence will be inserted between the pairs of double barcodes, separating them (Figures 3H and 3I). Set up a Golden Gate assembly reaction:

**Table:** 

Component	Final amount
Vector – 1916 bp	400 ng
Insert – 3206 bp	600 ng (equimolar to vector)
T4 ligase buffer	6 μL
T4 ligase	1.2 μL
BbsI	1.2 μL
ddH_2_O	To 60 μL

Include a negative control without the insert.

    ***Note:*** This cloning step could be completed as a traditional restriction-ligation as before, but Golden Gate cycling increases cloning efficiency to preserve barcode diversity.
  d. Run the following Golden Gate assembly program in a thermocycler with a heated lid:

**Table:** 

Steps	Temperature	Time	Cycles
Restriction	37°C	2 min	30
Ligation	16°C	5 min	
Enzyme inactivation	80°C	20 min	1
  e. Remove the sample from the thermocycler and let it cool down to 20°C–25°C.
  f. Add 0.5 μL of BbsI to the reaction. Mix well by flicking the tube, centrifuge briefly.
  g. Incubate 1h at 37°C in a thermocycler with a heated lid.
    ***Note:*** Additional digestion with BbsI after Golden Gate cycling is necessary to remove the empty vector background.
  h. Heat-inactivate BbsI by incubating 20 min at 80°C in a thermocycler with a heated lid.
  i. Desalt the ligation reaction as described in step 1g.
  j. Transform the desalted reaction into electrocompetent *E. coli* as described in step 1h.
  k. Plate serial dilutions of recovered cells to estimate transformation efficiency as described in 1i.
  l. Inoculate cultures of transformed library as described in step 1j.
  m. Estimate transformation efficiency as described in 1k and double barcode diversity.
    ***Note:*** In this step, expect the background to be 5000 colonies or less.
  n. Isolate plasmid DNA from the 50 mL culture using PureYield Plasmid Midiprep System (Promega) or equivalent.
4. Assemble the insulator-seq acceptor library.
  a. Inoculate insulator-seq reporter vector-carrying bacteria (Addgene 221460) into LB with selective antibiotic (100 μg/mL ampicillin) and incubate at 37°C 16–18 h with vigorous shaking in a bacterial incubator.
  b. Isolate plasmid DNA from the *E. coli* cultures using PureYield Plasmid Midiprep System (Promega) or equivalent.
  c. The double barcode library with the 3′UTR-Enhancer-Neutral DNA-3′UTR insert obtained in the previous step is going to serve as an insert, and gypsy-eGFP-Neutral DNA-mCherry-hsp70-Neutral DNA plasmid as a vector for Golden Gate assembly (Figures 3J and 3K). Set up a Golden Gate assembly reaction:

**Table:** 

Component	Final amount
Vector – 6416 bp	600 ng
Insert −2965 bp	280 ng (equimolar to vector)
T4 ligase buffer	6 μL
T4 ligase	1.2 μL
BsaI-HF-v2	1.2 μL
ddH_2_O	To 60 μL

Include a negative control without the insert.
  d. Run the following Golden Gate assembly program in a thermocycler with a heated lid:

**Table:** 

Steps	Temperature	Time	Cycles
Restriction	37°C	2 min	30
Ligation	16°C	5 min	
Enzyme inactivation	80°C	10 min	1
  e. Remove the sample from the thermocycler and let cool it down to 20°C–25°C.
  f. Add 0.5 μL of BsaI-HF-v2 to the reaction. Mix well by flicking the tube, centrifuge briefly.
  g. Incubate 1h at 37°C in a thermocycler with a heated lid.
  h. Heat-inactivate BsaI-HF-v2 by incubating 20 min at 80°C in a thermocycler with a heated lid.
  i. Desalt the ligation reaction as described in step 1g.
  j. Transform the desalted reaction into electrocompetent *E. coli* as described in step 1h.
  k. Plate serial dilutions of recovered cells to estimate transformation efficiency as described in step 1i.
  l. Inoculate cultures of transformed library as described in step 1j.
  m. Estimate transformation efficiency as described in step 1k and double barcode diversity.
    ***Note:*** The number of colonies obtained in this step reflects the maximum possible diversity of the acceptor library. However, the real number of unique double barcode combinations in the acceptor library pool is lower than the number of colonies obtained in the last cloning step.
  n. Isolate plasmid DNA from the 50 mL culture using PureYield Plasmid Midiprep System (Promega) or equivalent.
5. Generate an insulator-seq test fragment pool by DNA tagmentation.
  ***Note:*** Depending on the aim of the study, randomly fragmented genomic DNA fragments (Figure 3L) or synthetic oligo pools (Figure 3M) can be used as test fragments for insulator-seq. In this step we provide a protocol for tagmenting genomic DNA for insulator-seq. If your insert is an oligo pool, you can skip this step and move to step 6.
  a. Isolate BAC DNA from the *E. coli* cultures. For best results, use an ion-exchange column with a midi- or maxiprep kit, for example a QIAGEN Plasmid Midi Kit (QIAGEN).
    **CRITICAL:** Resuspend DNA that will be tagmented in 10 mM Tris-HCl pH 8.5 without EDTA.
  b. Purify Tn5 as described in the published step-by-step protocol.^6^
  c. Prepare Tn5 adapters.
    i. Resuspend lyophilized oligos (Tn5ME rv, Tn5ME-GibsonL15, Tn5ME-GibsonR15 – see Table 1 for sequences) in annealing buffer (50 mM NaCl, 40 mM Tris-HCl pH 8) at 200 μM final concentrations.
    ii. Mix equal volumes of Tn5ME rv and Tn5ME-GibsonL15 (adapter pair I) or Tn5ME-GibsonR15 (adapter pair II) in separate tubes.
    iii. Split the adapter pair mixes into 10 μL aliquots.
    iv. Anneal the oligos in a thermocycler with a heated lid using the following programme:

**Table:** 

Steps	Temperature	Time	Cycles
Denaturation	95°C	3 min	1
Incubate at Tm	65°C	5 min	1
Cool down	−0.5°C	15 s	140
Hold	10°C	forever	–

      **Pause point:** Annealed adapters can be stored at −20°C. Avoid freezing and thawing adapter aliquots more than 2 times.
  d. Load Tn5 with custom adapters.
    i. Set up a reaction on ice:

**Table:** 

Component	Final amount
Annealed adapter pair I	5 μM
Annealed adapter pair II	5 μM
Tn5	3.8 μM
Nuclease-free H_2_O	To 10 μL
    ii. Mix well by pipetting up and down.
      **CRITICAL:** Efficient mixing throughout the tagmentation protocol is required.
    iii. Incubate 30 min at 23°C in a thermocycler.
    iv. Place the tube with loaded Tn5 on ice.
      **CRITICAL:** Do not exceed the suggested Tn5 loading time. Prepare buffers and reaction used in subsequent steps before loading the enzyme.
  e. Tagment DNA.
    i. Dilute BAC DNA to 60 ng/μL.
    ii. Dilute loaded Tn5 by adding 90 μL of ice-cold nuclease-free H_2_O and mix by pipetting.
    iii. Assemble tagmentation reaction master mix (final total volume – 180 μL) on ice:

**Table:** 

Component	Final concentration	Amount for 180 μL total
Diluted loaded Tn5	–	99 μL
TAPSO-NaOH pH 8.2	10 mM	–
MgCl_2_	5 mM	–
PEG8000	8%	–

      ***Note:*** We recommend making a master mix using most of the loaded Tn5 because some of the components of the tagmentation reaction are viscous and are difficult to efficiently resuspend in small volumes.
    iv. Mix well by pipetting up and down.
    v. Distribute 9 μL of master mix into clean tubes.
    vi. Add 1 μL of BAC DNA (60 ng). Mix well by flicking the tubes.
      ***Note:*** We recommend setting up individual tagmentation reactions for each BAC used in the assay.
    vii. Transfer tubes from ice to a thermocycler pre-heated to 55°C.
    viii. Incubate at 55°C for 10 min.
    ix. To each 10 μL tagmentation reaction, add 2.5 μL of freshly diluted 0.2% SDS. Flick the tubes to mix. Do not vortex, this will generate foam.
    x. Incubate at 80°C for 10 min to inactivate Tn5.
    xi. Add 12.5 μL of nuclease-free H_2_O.
      **Pause point:** Tagmented DNA can be stored for several days at 4°C.
  f. PCR-amplify tagmented DNA.
    **CRITICAL:** Do not use HotStart DNA polymerase for this step.
    i. Set up a PCR reaction on ice using components from the KAPA HiFi PCR kit:

**Table:** 

Reagent	Final concentration/amount
KAPA 5× Buffer	10 μL
dNTPs	0.3 mM
KAPA HiFi Pol	1.3 μL
Primer 1 (Tn5ME-GibsonL PCR)	0.8 μM
Primer 2 (Tn5ME-GibsonR PCR)	0.8 μM
Tagmented DNA	9.6 ng
ddH_2_O	To 50 μL
    ii. Run the PCR program in a thermocycler with a heated lid:

**Table:** 

Steps	Temperature	Time	Cycles
Gap-filling	72°C	3 min	1
Initial denaturation	98°C	30 s	1
Denaturation	98°C	30 s	16
Annealing	58°C	20 s	
Extension	72°C	30 s	
Final extension	72°C	2 min	1
  g. Gel-purify the PCR product.
    i. Load the sample into a well of 1% TBE agarose gel and migrate for 15–30 min (Figure 5).

**Figure 5:**
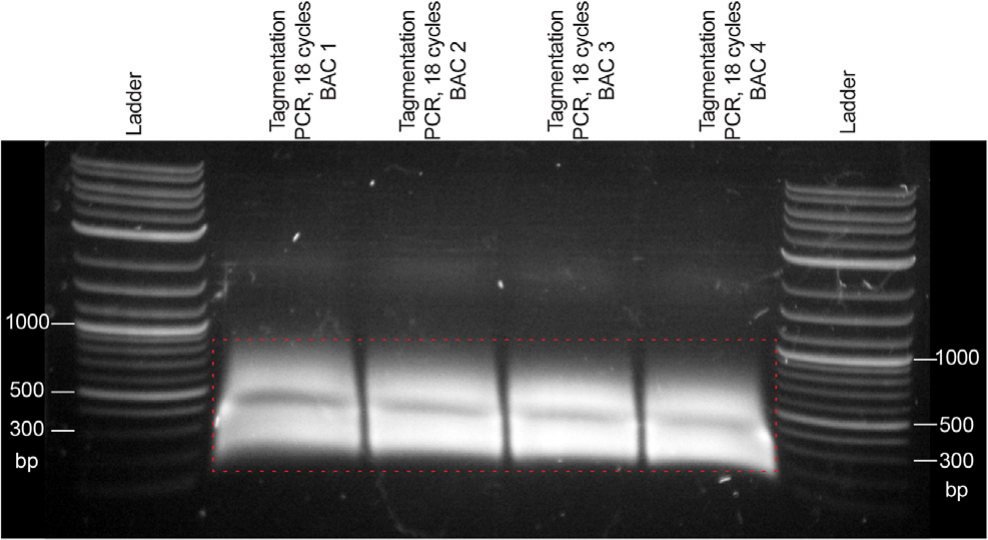
Tagmentation PCR on the gel Tagmentation PCR after 16 cycles of amplification visualized on a 1% agarose-TBE gel after 30 min of electrophoresis. DNA size ladder is labeled. Gel fragment outlined by the red dashed lines were excised from the gel and purified.
    ii. Excise fragments in the desired size range (100–900 bp in this study) from the gel.
    iii. Extract DNA from the TBE agarose gel using a NucleoSpin Gel and PCR Cleanup Kit (Macherey-Nagel) or equivalent.
      Troubleshooting 3**:** No PCR product after tagmentation.
      **Pause point:** Purified PCR product can be stored at −20°C.
6. If an oligo pool is used instead of tagmented BACs, generate an insulator-seq test fragment pool by amplifying an oligo pool.
  a. Design an insert library as described in the “before you begin” section.
  b. Amplify oligo pool library by PCR.
    i. Set up a reaction on ice:

**Table:** 

Reagent	Amount
DNA template	500 ng
2× KAPA mix	1×
Primer 1 (Gibson Homology M13 fw)	1 mM
Primer 2 (Gibson Homology M13 rv)	1 mM
ddH_2_O	to 500 μL
    ii. Mix by flicking the tube and centrifuge briefly.
    iii. Split the master mix into 10 tubes.
    iv. Run the PCR program in a thermocycler with a heated lid:

**Table:** 

Steps	Temperature	Time	Cycles
Initial Denaturation	98°C	45 s	1
Denaturation	98°C	15 s	8–10 cycles
Annealing	64°C	30 s	
Extension	72°C	15 s	
Final extension	72°C	1 min	1
  c. Purify the PCR product using MinElute PCR Cleanup Kit or equivalent as detailed in step 1b.
    ***Note:*** Spike-in controls (insulator reporter plasmids with neutral DNA or insulators of known strengths) can be amplified from genomic DNA with the same Gibson homology overhangs for low-throughput cloning.
    **Pause point:** Purified PCR product can be stored at −20°C.
7. Assemble the insulator-seq reporter library by Gibson assembly.
  a. Linearize the acceptor library (generated in step 4) with BamHI.
    i. Set up a restriction digestion reaction:

**Table:** 

Reagent	Amount
Acceptor library plasmid DNA	10 μg
10× rCutSmart Buffer (NEB)	100 μL
BamHI-HF (NEB)	10 μL
ddH_2_O	To 1000 μL
    ii. Incubate 3h at 37°C in a thermocycler with a heated lid.
    iii. Purify linearized DNA using a NucleoSpin Gel and PCR Cleanup Kit (Macherey-Nagel) or equivalent.
      ***Optional:*** Confirm that linearization was complete by digesting the linearized DNA with a second enzyme (for example, PciI) and visualizing the result on a 1% agarose TBE gel.
  b. Assemble the final library by Gibson assembly (Figure 3N).
    i. Set up the Gibson assembly reaction:

**Table:** 

Reagent	Final concentration/amount
Vector – 7000 bp	500 ng
Insert ∼500 bp	60 ng (2× molar excess)
Gibson mix (NEB)	30 μL
ddH_2_O	To 60 μL
    ii. Transfer to thermocycler pre-heated to 50°C.
    iii. Incubate 30 min at 50°C.
    iv. Immediately transfer the tubes on ice.
      **CRITICAL:** The Gibson assembly reaction must be transferred directly from ice to the pre-heated incubator and vice versa at the end of the reaction.
  c. Desalt the assembly reaction as described in step 1g.
  d. Transform the desalted reaction into electrocompetent *E. coli* as described in step 1h.
  e. Plate serial dilutions of recovered cells to estimate transformation efficiency as described in 1i.
  f. Inoculate cultures of transformed library as described in 1j.
  g. Estimate transformation efficiency as described in 1k and the library complexity.
    ***Note:*** In this step, the number of obtained colonies reflects the maximum number of unique double-barcode and insert combinations. While estimating the diversity of the acceptor and reporter library it is important to remember that it is a rough approximation, and we find the diversity of the reporter library that we estimate after sequencing to be approximately half of that expected from colony count. We find that a proportion of barcodes in the DNA-seq is always ambiguous, meaning associated with multiple test fragments. The proportion of ambiguous barcodes depends on the relative diversities of test fragments and reporter acceptor plasmids. Ambiguous barcodes must be removed from analysis computationally during the data preprocessing.
  h. Isolate plasmid DNA from the 50 mL culture using a PureYield Plasmid Midiprep System (Promega) or equivalent. Transfection-grade plasmid DNA is critical for the next step.

### Insulator-seq

**Timing: 4 days**

**Figure 6:**
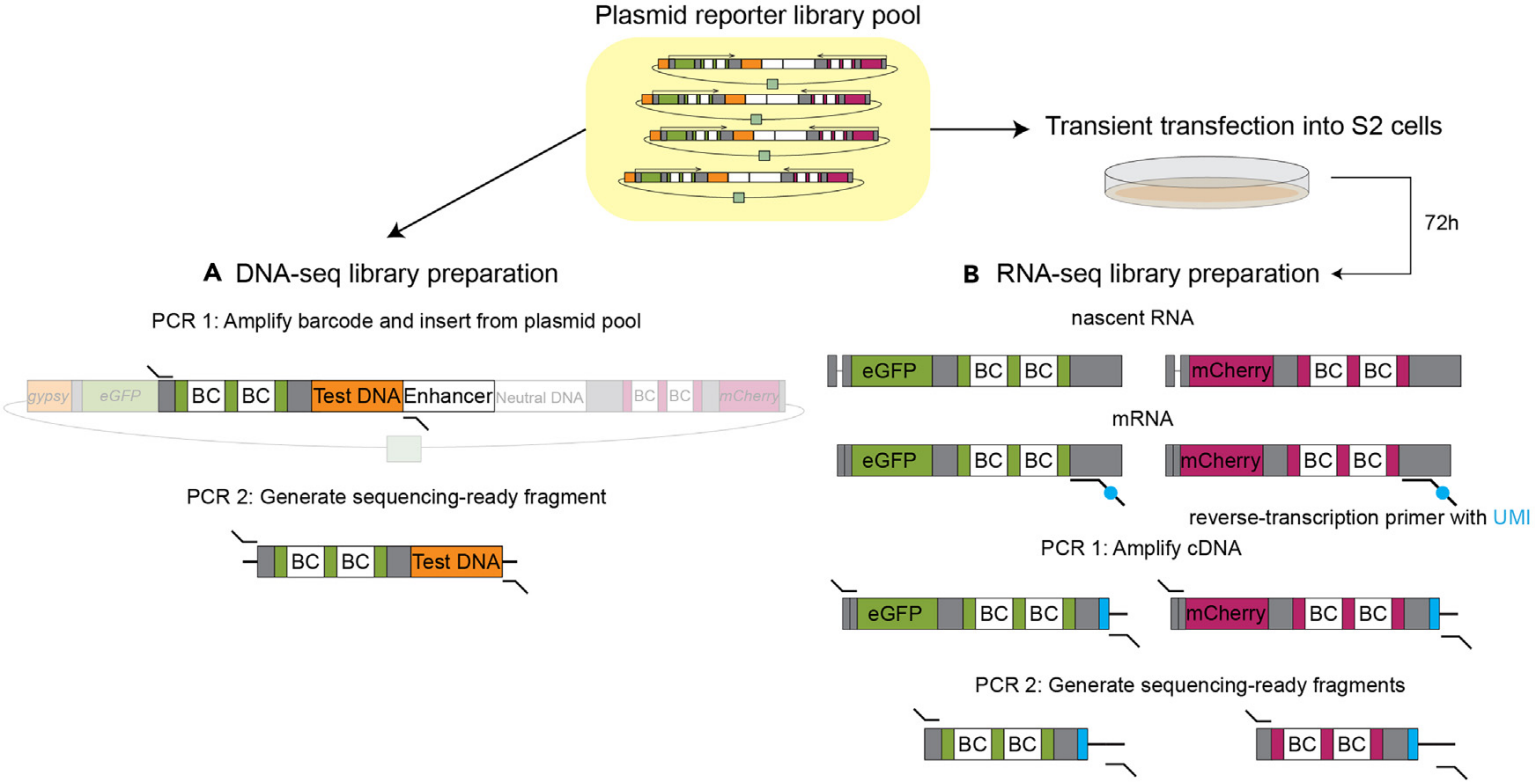
Insulator-seq library preparation overview (modified from Tonelli et al.) (A) DNA-seq library preparation from a plasmid pool (step 13). (B) RNA-seq library preparation from total RNA of transfected S2R + *Drosophila* cells (step 14).

The insulator-seq reporter library is transiently transfected into *Drosophila* S2R + cells, followed by total RNA extraction (Figure 6).

8. Seed cells a day before transfection.
  a. Grow *Drosophila* S2R + cells (Drosophila Genomics Resource Center stock number 150) in Schneider medium supplemented with 10% heat-inactivated FBS and 1% penicillin-streptomycin at 25°C until confluent in a 10 cm dish.
  b. Detach cells from the dish by continuously aspirating and pipetting the medium over the cells.
  c. Collect the cells in a Falcon tube.
  d. Estimate cell density using a hemocytometer or other method.
  e. Seed 25 million cells into a 10 cm dish in a final volume of 10 mL. Prepare biological duplicates for each distinct insulator-seq plasmid library.
9. Transfect the cells with insulator-seq plasmid library using Effectene transfection reagent (QIAGEN).
  **CRITICAL:** Include spike-in controls at this step. We recommend that the spike-in plasmids constitute 0.1% of total transfected DNA library pool. We added. 5 ng of each of 4 spike-in plasmids (20 ng total) per 2 μg of insulator reporter plasmid library.
  a. Dilute 2 μg of plasmid library DNA and 20 ng of spike-in control plasmid DNA with EC buffer to a final volume of 300 μL.
  b. Add 16 μL of Enhancer.
  c. Vortex for 5 s. Centrifuge briefly.
  d. Incubate 5 min at 20°C–25°C.
  e. Add 60 μL of Effectene.
  f. Vortex for 10 s. Centrifuge briefly.
  g. Incubate 10 min at 20°C–25°C.
  h. Remove culture medium from the dish, avoiding detaching the cells. Replace with 7 mL of fresh medium, pre-warmed to 25°C.
  i. Add 3 mL of fresh medium to the transfection mix, pipette up and down twice and immediately add to the cells dropwise, while gently swirling the dish.
  j. Incubate for 72 h.
10. Harvest cells and isolate total RNA.
  a. Confirm that the transfection was successful.
    i. Confirm that the cells are transfected (green and red fluorescent) using an epifluorescence microscope.
      ***Optional:*** Estimate transfection efficiency by analyzing a small aliquot of cells on a fluorescence-activated cell sorter or analyzer.
  b. Transfer the cells into a 15 mL tube.
  c. Sediment the cells by centrifugation at 2000 g for 10 min.
  d. Discard the cell culture supernatant.
  e. Extract total RNA by phenol-chloroform extraction followed by isopropanol precipitation.
    ***Note:*** Column-based kits can be used in this step as an alternative.
    **CRITICAL:** Avoid RNase contamination by following good laboratory practice when working with RNA.
    i. Dilute 750 μL TRIzol LS reagent (Thermo Fisher Scientific) with 250 μL nuclease-free H_2_O per sample.
    ii. Add 1 mL of 0.75× TRIzol to the cell pellet.
    iii. Resuspend the cell pellet by pipetting up and down until the pellet is dissolved.
    iv. Incubate 5 min at 20°C–25°C.
    v. Centrifuge 12000 g for 10 min at 4°C.
    vi. Transfer the supernatant to a new 1.5 mL DNA Lo-Bind tube.
    vii. Add 200 μL chloroform.
    viii. Vigorously shake the tubes for 30 s.
    ix. Incubate 10 min at 20°C–25°C.
    x. Centrifuge 12000 g for 15 min at 4°C.
    xi. Transfer the upper aqueous phase (approximately 480 μL) to a new tube.
    xii. Precipitate RNA by adding 500 μL isopropanol and mixing by inversion.
    xiii. Incubate at 20°C–25°C for 10 min.
    xiv. Centrifuge 12000 g for 10 min at 4°C.
  f. Wash RNA pellet with EtOH.
    i. Discard supernatant from the previous step.
    ii. Wash pellet with 1 mL freshly prepared 80% EtOH in nuclease-free water.
    iii. Flick the tube to mix.
    iv. Centrifuge 12000 g for 5 min at 4°C.
    v. Repeat the wash (two washes total).
    vi. After the last wash, remove residual ethanol.
    vii. Air-dry the pellet at 20°C–25°C for 5 min.
  g. Resuspend the RNA pellet in 44 μL nuclease-free water.
11. Treat the extracted RNA with DNase.
  a. Assemble the reaction:

**Table:** 

Reagent	Final amount
Total RNA	44 μL
10× Buffer	5 μL
Turbo DNase (Thermo Fisher Scientific)	1 μL
  b. Incubate 30 min at 37°C.
12. Purify RNA with RNAClean XP beads.
  a. Vortex the beads.
  b. Add 1.8 vol of beads (90 μL) to the sample (50 μL).
  c. Mix by pipetting up and down 15× (vortexing is not recommended).
  d. Incubate on ice for 15 min.
  e. Place the tube on a magnetic tube stand and incubate 5 min.
  f. Remove supernatant, avoid touching the beads.
  g. Without removing the tubes from the stand, wash the beads 3 times with 1 mL freshly prepared 75% EtOH in nuclease-free water. Incubate with 75% EtOH for 30 s during each wash.
  h. Briefly centrifuge the tube after the last wash. Place the tube on the magnetic tube stand.
  i. Remove residual ethanol.
  j. Air-dry the beads for 2 min.
    **CRITICAL:** Do not over-dry the beads. They should remain glossy. Over-drying leads to poor recovery.
  k. Resuspend the beads in 30 μL of nuclease-free water.
  l. Place the tube on the magnetic tube stand for 2 min.
  m. Aspirate eluted RNA, avoid aspirating the beads.
  n. Measure the RNA concentration with Qubit HS RNA kit or equivalent. Expect 150 μg total RNA per 10 cm dish.
    **Pause point:** RNA can be aliquoted, snap-frozen in liquid nitrogen and stored at −80°C.

### Preparation of insulator-seq sequencing libraries

**Timing: 2–3 days**

This step details library preparation for DNA-seq, that will link barcodes in reporter genes with test fragments, and RNA-seq, which will quantify insulator strengths of test fragments (Figure 6). We describe the PCR conditions and number of cycles that were used to make libraries in this study; however, these might require optimization depending on the experiment.

13. Make DNA-seq library.
  a. First PCR: amplify barcode and insert from the plasmid pool.
    i. Set up a PCR reaction on ice:

**Table:** 

Reagent	Final concentration/amount
2× KAPA mix (Roche)	25 μL
DNA PCR1 fw	0.8 μM
DNA PCR1 rv	0.8 μM
Plasmid DNA	10 ng
ddH_2_O	To 50 μL
    ii. Run the following PCR program in a thermocycler with a heated lid:

**Table:** 

Steps	Temperature	Time	Cycles
Initial denaturation	98°C	45 s	1
Denaturation	98°C	10 s	10 cycles
Annealing	59°C	20 s	
Extension	72°C	30 s	
Final extension	72°C	2 min	1
Hold	4°C	Forever
  b. Purify the PCR 1 product on SPRIselect beads (Beckman-Coulter).
    i. Vortex beads to resuspend.
    ii. Add 1.8 vol of beads (90 μL) to the sample (50 μL).
    iii. Mix well by pipetting up and down or vortexing.
    iv. Incubate 5 min at 20°C–25°C.
    v. Place the tube on magnetic tube stand. Incubate 5 min.
    vi. Discard supernatant.
    vii. Without removing the tube from the magnetic stand, wash twice with 80% freshly prepared EtOH, incubating each wash for 30 s.
    viii. After discarding the last wash, centrifuge the tube briefly and place back on the magnetic stand. Allow beads to separate and then remove the residual ethanol without disturbing the beads.
    ix. Air-dry for 2–3 min.
    x. Resuspend the beads in 20 μL elution buffer (10 mM Tris-HCl pH 8.5) and incubate 2 min at 20°C–25°C.
    xi. Place the tube on the magnetic rack for 5 min.
    xii. Collect the eluted DNA in a clean 1.5 mL DNA LoBind tube. Avoid aspirating the beads.
    xiii. Measure DNA concentration with Qubit DNA HS Kit or equivalent. Expect 2-8-fold amplification of the input DNA.
  c. Second PCR: add Illumina adapters.
    i. Set up a reaction on ice.
      **CRITICAL:** Remember to use primers with different Illumina barcodes for each independent plasmid library.

**Table:** 

Reagent	Final concentration/amount
2× KAPA mix (Roche)	25 μL
DNA PCR2 fw	0.8 μM
DNA PCR2 rv	0.8 μM
PCR 1 product	15 μL
ddH_2_O	To 50 μL
    ii. Run the following PCR program in a thermocycler with a heated lid:

**Table:** 

Steps	Temperature	Time	Cycles
Initial denaturation	98°C	45 s	1
Denaturation	98°C	10 s	5 cycles
Annealing	68°C	20 s	
Extension	72°C	30 s	
Final extension	72°C	2 min	1
Hold	4°C	forever
  d. Purify the PCR 2 product on SPRIselect beads (Beckman-Coulter) using 1 vol of beads following the procedure described above. Expect 5-15-fold amplification of the template DNA.
  e. Confirm the size distribution of the library is as expected by using a Bioanalyzer or a Fragment Analyzer (Agilent) (Figure 7).

**Figure 7:**
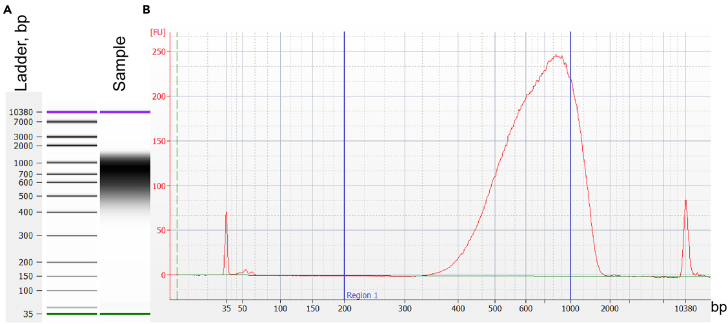
Bionalyzer trace showing size distribution of the DNA PCR2 product (A) Bioanalyzer trace represented as a gel. (B) Bioanalyzer trace represented as an electropherogram. Blue vertical lines show the optimal size range.
  f. Remove potential primer dimers and fragments >1 kb by performing an additional SPRIselect cleanup according to the manufacturer’s instructions.
  g. Make an equimolar pool of samples to be sent for sequencing according to instructions given by the company.
    Troubleshooting 4**:** No amplification, low DNA concentration, or size distribution is not as expected.
14. Make RNA-seq library.
  a. Synthesize cDNA from total RNA using reporter gene-specific primers.
    **CRITICAL:** To obtain more accurate counts of reporter gene mRNAs, we strongly recommend using the barcoded reverse transcription primer (Table 1) with unique molecular identifiers (UMIs), which will allow you to distinguish between PCR multiplications and unique mRNA molecules.
    i. Set up a reaction on ice:

**Table:** 

Reagent	Final concentration/amount
2 μM gene-specific primer with UMI	1 μL
10 mM dNTP mix	1 μL
Total RNA	5 μg
Nuclease-free water	To 13 μL
    ii. Mix and briefly centrifuge the tube.
    iii. Incubate at 65°C for 5 min to anneal the primer to RNA, transfer the tube to ice and incubate for at least 1 min.
    iv. Set up reverse transcription reaction mix on ice:

**Table:** 

Reagent	Final concentration/ amount
5× SSIV Buffer	4 μL
100 mM DTT	1 μL
RNaseOUT Recombinant RNase Inhibitor	1 μL
Superscript IV reverse transcriptase (Thermo Fischer)	1 μL
    v. Add reverse transcription reaction mix (7 μL) to the annealed RNA (13 μL). Mix by pipetting up and down.
    vi. Incubate 1 h at 52°C.
    vii. Heat-inactivate the reaction – incubate 10 min at 80°C.
      **Pause point:** cDNA can be stored at −20°C.
  b. First PCR to amplify reporter gene cDNA.
    ***Note:*** The first PCR primer (RNA PCR 1) anneals to spliced *eGFP* and *mCherry* cDNA and must always be used to avoid amplifying plasmid DNA that remains despite the DNase treatment. The choice of the reverse primer depends on whether the reverse transcriptase primer with or without UMI was used. The same reverse transcription primer can be used for making libraries without UMIs, while DNA PCR 2 fw barcoded primer must be used for UMI libraries.
    i. Set up a reaction on ice:

**Table:** 

Reagent	Final concentration/amount
2× KAPA mix (Roche)	50 μL
RNA PCR 1	0.8 μM
DNA/RNA PCR 2 fw	0.8 μM
cDNA	5 μL
ddH_2_O	To 100 μL
    ii. Run the following PCR program in a thermocycler with a heated lid:

**Table:** 

Steps	Temperature	Time	Cycles
Initial denaturation	98°C	45 s	1
Denaturation	98°C	10 s	15 cycles
Annealing	65°C	20 s	
Extension	72°C	45 s	
Final extension	72°C	2 min	1
Hold	4°C	Forever
    iii. Purify the PCR product using MinElute PCR purification kit as described in step 1b. Elute in 20 μL elution buffer (10 mM Tris-HCl pH 8.5).
    iv. Quantify the DNA concentration and determine the yield of the PCR. Expect between 40 and 200 ng total yield.
      Troubleshooting 5**:** No amplification after first PCR.
  c. Second PCR to amplify gene barcodes and add Illumina adapters.
    **CRITICAL:** Use the same DNA/RNA PCR 2 fw primers for the same samples as in the first RNA-seq PCR.
    i. Set up a reaction on ice:

**Table:** 

Reagent	Concentration (stock)	Final concentration	Amount, μl
2× KAPA mix (Roche)	2×	1×	25 μL
DNA/RNA PCR 2 fw	10 μM	0.8 μM	4 μL
DNA/RNA PCR 2 rv	10 μM	0.8 μM	4 μL
PCR 1 product	—	—	15 μL
ddH_2_O	—	—	2 μL
    ii. Run the following PCR program in a thermocycler with a heated lid:

**Table:** 

Steps	Temperature	Time	Cycles
Initial denaturation	98°C	45 s	1
Denaturation	98°C	10 s	6 cycles
Annealing	68°C	20 s	
Extension	72°C	15 s	
Final extension	72°C	1 min	1
Hold	4°C	Forever
  d. Purify the second PCR product on SPRIselect beads using the right-sided size selection protocol.
    i. Vortex beads to resuspend.
    ii. Add 0.6 vol of beads (30 μL) to the sample (50 μL).
    iii. Mix well by pipetting up and down or vortex.
    iv. Incubate 5 min at 20°C–25°C.
    v. Place the tube on a magnetic tube stand. Incubate 5 min.
    vi. Collect supernatant into a clean 1.5 mL DNA LoBind tube.
    vii. Add 1.2 vol of beads (60 μL) to the supernatant.
    viii. Continue the purification as described for the left-sided size selection protocol in step 13b.
    ix. Measure the DNA concentration with Qubit DNA HS Kit or equivalent. Expect a 2-6-fold amplification of the input DNA.
  e. Confirm the size distribution of the RNA-seq library Using a Bioanalyzer or Fragment Analyzer (Agilent) (Figure 8).

**Figure 8:**
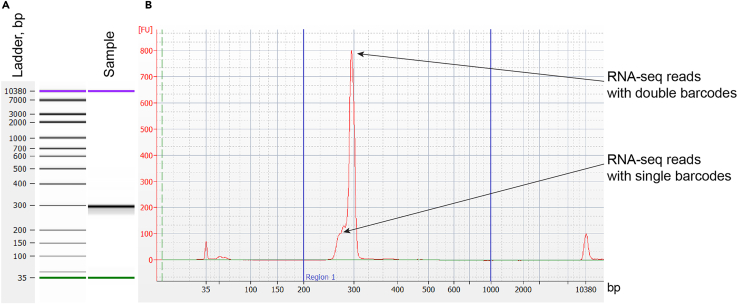
RNA-seq Bioanalyzer trace (A) Bioanalyzer trace represented as a gel. (B) Bioanalyzer trace represented as an electropherogram. Arrows indicate RNA-seq fragments that have a single or a double barcode. Blue vertical lines show the optimal size range.
  f. Remove potential primer dimers and fragments >1 kb by performing an additional SPRIselect cleanup according to the manufacturer’s instructions.
  g. Make an equimolar pool of samples for Illumina sequencing according to the sequence facility’s instructions.

### Data analysis

**Timing: 1–5 days**

Scripts used for pre-processing insulator-seq data with and without UMI are available in our repository on GitHub.^5^ Here, we provide an example of a simple data analysis followed by visualization in the genome browser.

15. Pre-process DNA and RNA-seq data and merge the two datasets together.
  Follow the instructions provided on GitHub to complete this step. We recommend performing data pre-processing on a cluster or high-performance Linux server.
  a. Go to the GitHub repository as indicated in ref.^5^ and download the files and scripts for the type of assay performed (insulator-seq on genomic DNA or on synthetic DNA), as well as the training data with GEO accession numbers.
  b. Execute “BAC_screen.sh” or “Synthetic_screen.sh” scripts based on the type of assay.
  c. Obtain a table that has the following structure:

**Table:** 

Chromosome	Start	End	Barcode	mCherry	eGFP	Ratio
16. Bin BAC insulator-seq data into bins of the desired size.
  ***Note:*** We found that assigning an insulator strength to a genomic bin by taking the median insulator score of all fragments that overlap the bin is the most robust way of visualizing insulator-seq data.
  a. Generate 50 bp genomic bins using a reference genome chromosome list: bedtools makewindows -g chrom_list.txt -w 100 > 50bp_bins.bed.
  b. Assign an insulator score to the bin:
    bedtools map -a 50bp_bins.bed -b final_table.bed -c 7 -o median -null 0 > 50bp_median.bedgraph
    ***Note:*** In the case of insulator-seq on synthetic DNA fragments, use the oligo pool library as bins and assign the median insulator strength of all independently cloned versions (with different barcodes) of a given synthetic DNA fragment to each synthesized fragment instead.
17. Visualize the result by loading it into IGV or utilize the Gviz Bioconductor package.^10^

## Expected outcomes

Insulator-seq identifies regions with insulator activity in a high-throughput, unbiased way. The major outcome of the assay is a quantitative assessment of insulator activities of test DNA fragments obtained through tagmentation of genomic DNA or by DNA synthesis. With insulator-seq, we were able to recover known insulators of abdominal *HOX* genes in the *Drosophila bithorax complex* (*BX-C*) and to discover insulators in another locus. Using an oligo pool library of systematically mutated versions of CTCF-dependent insulators, we were able to determine that additional DNA sequences apart from the CTCF motif are critical for insulation.

## Limitations

Insulator-seq library barcoding has certain limitations. During data analysis we discovered that up to half of transcribed *eGFP* barcodes in the RNA-seq data do not have a matching transcribed *mCherry* barcode. This suggests that half of the cloned insulator reporter acceptor plasmids do not carry matching barcodes in *eGFP* and *mCherry*. In the future, using emulsion PCR for amplifying barcode oligo pool or increasing the spacing between the barcodes in an oligo may prevent such cloning limitations.

Insulator-seq can be costly if performed at large scale, as for a reporter library with estimated 2 million reporters (by colony count), we recommend sequencing 10 million DNA-seq reads and 200 million reads for each RNA-seq replicate.

In contrast to single reporter MPRA strategies, our dual reporter gene strategy does not require normalization of reporter gene expression by the relative abundances of insulator reporter plasmids containing different test fragments. Future improvements of insulator-seq could involve implementing normalizing the RNA-seq reads by the relative input abundance to enable the simultaneous discovery of insulators, enhancers and silencers in the same experiment.

The molecular mechanism of insulation on transiently transfected plasmids remains unknown. It is possible that certain genomic features can exert gene insulation in a chromosomal context but not in a transiently transfected plasmid assay, and will thus be missed by insulator-seq. Conversely, new genomic features discovered as insulators by insulator-seq should be functionally validated *in vivo*.

## Troubleshooting

### Problem 1

An arc appears during electroporation (related to step 1-h).

### Potential solution

An arc during electroporation is formed when the current passes outside of the cuvette, which might be caused by different reasons. It is important to have low salt concentration of the electroporated sample. Longer incubation times during agarose filtration (step 1f) might be necessary to remove salts from the cloning reaction but should not exceed 90 min. In case of longer desalting times we recommend doubling the volume of the cloning reactions, as incubation on agarose gel for long periods of time leads to loss of sample volume.

When electroporating, it is critical that the volume of bacteria and cloning reaction mix is 1 mm below the metal part of the cuvette, and never fills the chamber completely. Adjust the suggested volume of bacteria and cloning reaction mix.

When mixing bacteria and cloning reaction mix, avoid introducing air bubbles and especially avoid bubbles collecting at the top of the cuvette. If the bubbles were created during mixing, transfer only the part of the mix where there are no bubbles into the electroporation cuvette and leave the bubbles in the tube in which the mixing occurred.

### Problem 2

Low transformation efficiency or no colonies at all (related to step 1k).

### Potential solution

In parallel to electroporation, also transform the cloning reaction and the negative control into chemically competent *E. coli* to determine whether the cloning reaction failed or whether the electroporation failed.

Be precise when preparing electrocompetent cells, regularly test them for competence using an undigested vector plasmid and ideally include a control using commercially available electrocompetent *E. coli*. We found home-made electrocompetent cells to be approximately 10 times less efficient for electroporation than commercial cells, which nevertheless provided us with enough clones to perform insulator-seq.

### Problem 3

No PCR product after tagmentation PCR or smeary bands.

### Potential solution

Tagmentation and subsequent PCR can fail for many reasons, including but not limited to: bad quality of the Tn5 enzyme, old annealed adapters, annealed adapter aliquots that were frozen and-thawed too many times, low quality of DNA template, or amplification with too few PCR cycles.

First, we recommend testing if the enzyme is active by performing tagmentation on plasmid DNA template using all of the loaded Tn5 and including a no Tn5 control. After tagmentation, the entire volumes of both reactions can be loaded on a 1% TBE-agarose gel and electrophoresed for 15 min. In the no enzyme control, the unfragmented DNA should be visible either as a single band or as two bands, showing circular and nicked plasmid DNA. In the reaction with Tn5 the DNA should not be visible and appear as a smear.

If the enzyme is confirmed active, for troubleshooting PCR, we recommend first to set up an end-point PCR (>= 30 cycles) using DNA that was either tagmented or incubated without Tn5 as negative control, and to load both PCR reactions on a 1% TBE-agarose gel, followed by electrophoresis for 30 min. The PCR-amplified tagmented DNA should appear as a smear ranging in size from 100 bp to approximately 2 kb, while no amplification or a faint smear might be visible in the no Tn5 control. After confirming that the PCR works, we recommend optimizing the number of the PCR cycles and choosing the minimal number of cycles where the PCR is sufficiently visible on the gel and allows for efficient size selection and gel extraction.

### Problem 4

No amplification, low DNA concentration, size distribution is not as expected, sharp peaks on Bioanalyzer.

### Potential solution

We suggest optimizing the number of PCR cycles, and selecting the minimum number of cycles that would yield a 2 to 8-fold amplification of the amount of template DNA in the first round of DNA library preparation.

In case the size distribution of PCR 2 is not as expected, confirm that the size selection on the gel during step 5 was correct. Optimize SPRI magnetic beads size selection during purification of PCR 1 and PCR 2 products by following the manufacturer’s instructions.

Sharp peaks on the Bioanalyzer might indicate primer dimers if the size is < 100 bp.

### Problem 5

No amplification after RNA PCR 1.

### Potential solution

The concentration of cDNA may vary based on the efficiency of RT-PCR reactions. Perform serial dilutions of cDNA to determine the optimum amount of template to use for RNA PCR 1. Confirm the quality of template RNA on the Bioanalyzer.

## Resource availability

### Lead contact

Further information and requests for resources and reagents should be directed to and will be fulfilled by the lead contact, Maria Cristina Gambetta (mariacristina.gambetta@unil.ch).

### Technical contact

Questions about the technical specifics of performing the protocol should be directed to the technical contact, Anastasiia Tonelli (anastasiia.tonelli@unil.ch).

### Materials availability

Plasmids generated in this study have been deposited to Addgene, as listed in the key resources table.

### Data and code availability

All original code is publicly available in GitHub.^5^

## Acknowledgments

This work was supported by the 10.13039/501100001711Swiss National Science Foundation (SNSF 219941 to M.C.G.), the 10.13039/501100023177Pierre Mercier Science Foundation (to M.C.G.), the Foundation for Research and Education in Genetics (FREG to A.T.), and the 10.13039/501100006390University of Lausanne.

## Author contributions

Conceptualization, A.T., P.C., and M.C.G.; methodology, A.T., P.C., and M.C.G.; investigation, A.T.; formal analysis, A.T. and P.C.; writing – original draft, A.T.; writing – review and editing, A.T. and M.C.G.; supervision, M.C.G.; funding acquisition, A.T. and M.C.G.

## Declaration of interests

The authors declare no competing interests.
